# Le carcinome neuro-endocrine cutané primitif: à propos d'un nouveau cas et revue de la littérature

**DOI:** 10.11604/pamj.2015.20.395.6149

**Published:** 2015-04-22

**Authors:** Samira Boukind, Oumkeltoum Elatiqi, Meriem Dlimi, Driss Elamrani, Yassine Benchamkha, Saloua Ettalbi

**Affiliations:** 1 Service de Chirurgie Plastique, Réparatrice, Esthétique et Brûlés, CHU Mohammed VI, Marrakech, Maroc

**Keywords:** Carcinome neuro-endocrine cutané primitif, ganglion sentinelle, traitement chirurgical, radiothérapie, surveillance, Cutaneous neuroendocrine carcinoma, sentinel lymph node, surgical treatment, radiotherapy, surveillance

## Abstract

Le carcinome neuro- endocrine cutané primitif (CNEC) est une tumeur cutanée rare et agressive du sujet âgé, favorisée par le soleil et l'immunodépression. Elle est caractérisée par une évolution agressive avec un fort taux de récidive, une évolution ganglionnaire régionale et un risque de métastases à distance. Nous rapportons un cas de cette tumeur chez un patient âgé de 67 ans sous forme d'un placard nodulaire hémorragique mesurant 16 /14 cm. Le patient a bénéficié d'une exérèse chirurgicale large avec couverture de la perte de substance par un lambeau musculo-cutané du muscle grand dorsal, un curage ganglionnaire axillaire et une radiothérapie adjuvante. Après un recul de 2 ans et 2 mois, le patient est toujours vivant sans métastase ni récidive. La littérature étant pauvre, la prise en charge diagnostique et thérapeutique est controversée et donc hétérogène. Globalement le pronostic est mauvais, et certains paramètres corrélés au pronostic sont précisés.

## Introduction

Les carcinomes neuro-endocrines cutanés (carcinome à cellules de Merkel) sont des tumeurs malignes cutanées rares développées au dépens des cellules neuro-endocrines cutanées, décrites initialement par Tocker en 1972 [1] sous le nom de carcinome trabéculaire de la peau. Le terme de Merkelome ou «tumeur à cellule de Merkel» est proposé en 1975 par Tang et Tocker [2] devant la présence de grains neurosécrétoires dans les cellules tumorales très proches, morphologiquement, de ceux observés dans les cellules de Merkel normales en microscopie électronique. Malgré la ressemblance indéniable entre la cellule de Merkel et les cellules des carcinomes neuroendocrines cutanés, aucun argument morphologique ni phytogénètique ne permet d'affirmer la filiation entre la cellule de Merkel normale et la néoplasie de telle sorte que l'origine du carcinome neuroendocrine cutané reste une énigme [2]. Ils surviennent préférentiellement chez les sujets âgés ou immunodéprimés et se localisent le plus souvent au niveau des zones photo exposées, notamment sur le visage [3]. Le cas de CNEC que nous rapportons, illustré par les données de la littérature, nous permettra de mettre le point sur cette rare tumeur cutanée.

## Patient et observation

**Figure 1:**
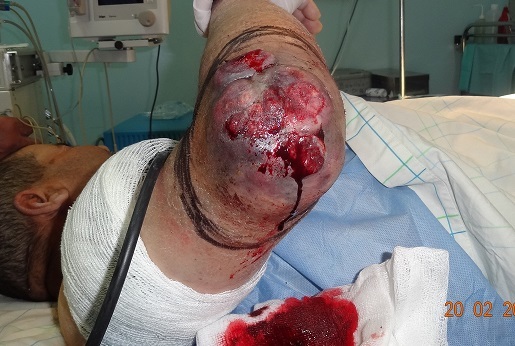
Aspect de la tumeur en pré- opératoire

**Figure 2:**
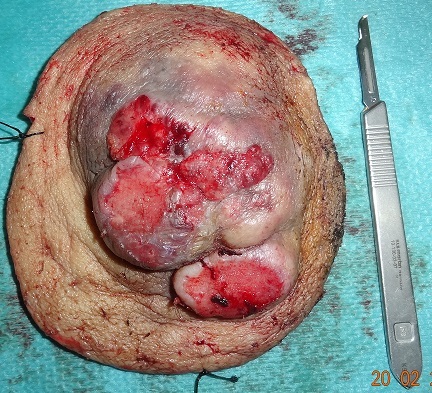
La pièce opératoire d'exérèse tumorale avec des marges latérales de 3 cm

**Figure 3:**
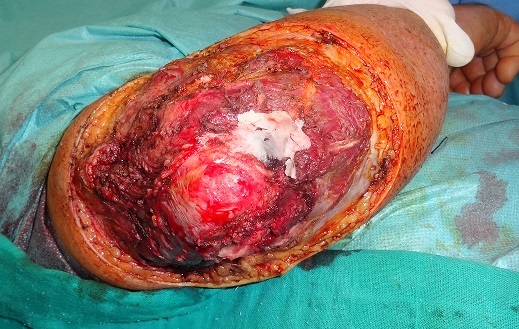
Aspect en per- opératoire de la perte de substance (PDS) post- exérèse tumorale, emportant l'aponévrose musculaire avec mise à nu de l'os

**Figure 4:**
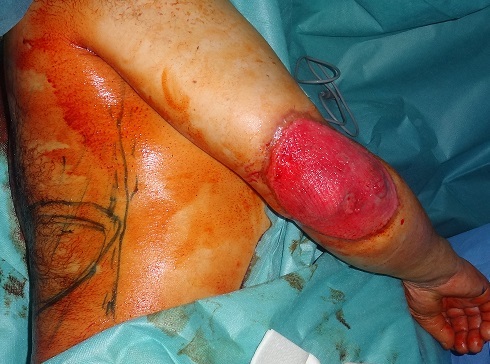
Le patient a bénéficié d'une reprise chirurgicale, vu la limite profonde tumorale, et une couverture de la PDS par un lambeau musculo- cutané du muscle grand dorsal

**Figure 5:**
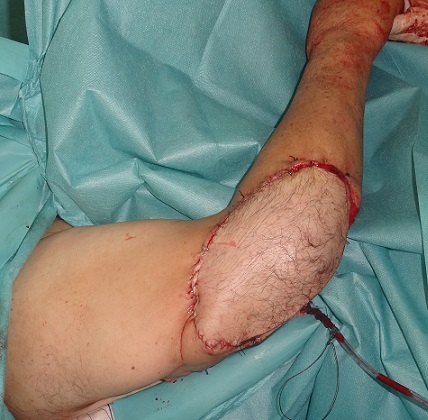
Aspect en post opératoire immédiat

**Figure 6:**
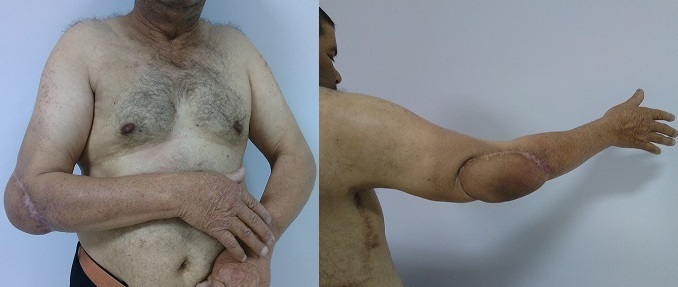
Aspect après 1 an et 9 mois

Patient âgé de 67 ans, sans antécédents notables, ayant consulté dans notre formation en Octobre 2012, pour une tumeur du coude droit apparu 6 mois auparavant, et qui a augmenté progressivement de volume. A l'examen, la masse est formé d'un placard nodulaire, de consistance dure, de coloration violacée, saignante au contact d'environ 16 /14 cm avec inflammation périphérique, mobile par rapport au plans sous jacents (Figure 1). Les aires ganglionnaires sont libres. L'analyse anatomopathologique d'une pièce de biopsie parlait d'une tumeur maligne indifférenciée à cellules rondes, et ulcérée en surface. Le diagnostic est confirmé par l’étude immuno-histochimique qui a conclu à un carcinome neuroendocrine malin de Merkel. Le patient a bénéficié, sous anesthésie générale, d'une exérèse chirurgicale large passant latéralement à 3 cm de la tumeur, et en profondeur emportant l'aponévrose musculaire (Figure 2, Figure 3). L'analyse histopathologique de la pièce retrouve les mêmes données de la biopsie et précise que les limites d'exérèses latérales sont saines passées entre 2 cm et 3 cm alors que la limite profonde était rasante. Egalement de nombreux emboles vasculaires étaient présents. A l'examen macroscopique, il s'agit d'une masse tumorale recouverte d'un lambeau cutané de 15/12 cm. A la coupe, il existe une tumeur mesurant 11/8,5/4,5 cm d'aspect blanc grisâtre et encéphaloide, parsemée de plages nécrotiques, elle ulcère la peau. En profondeur la tumeur infiltre le muscle. Une radiographie du coude était réalisée dans le cadre du bilan d'extension et qui n'a pas montré de lyse osseuse, ainsi qu'une TDM thoraco- abdomino- pelvienne et une échographie axillaire sans anomalie décelée notamment pas d'adénopathies. Le patient a bénéficié d'une reprise chirurgicale, vu la limite profonde tumorale, et une couverture de la perte de substance (mettant l'os à nu) par un lambeau musculo-cutané du muscle grand dorsal (Figure 4, Figure 5). Après cicatrisation du lambeau, une greffe de peau mince était réalisée au niveau de la face interne du bras et du coude pour couvrir la partie musculaire proximale du lambeau. L’évolution s'est marquée par l'apparition d'une adénopathie axillaire homolatérale mobile d'environ 2 cm de diamètre, 9 mois après le diagnostic de la tumeur. Un curage ganglionnaire axillaire était réalisé avec étude anatomopathologique ayant confirmé l'envahissement ganglionnaire (4 ganglions sur 21 sont positifs). Le patient a bénéficié d'une radiothérapie adjuvante, sur la zone d'exérèse tumorale et la zone du curage axillaire, à raison de 45 séances, avec une surveillance régulière, clinique à la recherche d'une récidive locale, en transit, ganglionnaire ou à distance. L'extension totale du coude était limitée à 160° (Figure 6). La surveillance paraclinique comportait un scanner thoraco- abdomino-pelvien et une IRM du coude droit, qui n'ont pas révélés d'anomalies. Après un recul de 2 ans et 2 mois le patient est toujours vivant sans récidive ni métastase.

## Discussion

Le carcinome à cellules de Merkel (CCM) est une tumeur cutanée appartenant au groupe des carcinomes neuroendocrines. C'est une tumeur rare [4]. Elle survient principalement chez le sujet âgé avec une moyenne d’âge de 60 ans (15-92 ans), aucun cas n'est décrit chez l'enfant. La tumeur intéresse quasi exclusivement les sujets de race blanche sans prédominance de sexe [2]. L'incidence réelle du CCM est inconnue, elle est évaluée à 0,45 cas pour 100000 en 2002 aux Etats- Unis [5]. Les principaux facteurs de risque de CCM sont l’âge avancé, l'exposition solaire et l'immunodépression [4]. Très récemment, il a été montré qu'un virus, désormais appelé *Merkel cell polyomavirus*, était impliqué dans la pathogénie du CCM [6], et était présent dans huit tumeurs de Merkel sur dix [5]. Il s'agit d'une tumeur non seulement agressive sur le plan locorégional, mais incluant surtout un risque élevé de métastases à distance. En effet, plusieurs revues ont évalué le risque de récidive locale entre 25 et 33% de tous les CCM, et le risque de métastases à distance de l'ordre de 33%. Le taux de mortalité du CCM est plus élevé que celui du mélanome, avec une survie à cinq ans allant de 30 à 64% [4]. Le CCM se présente cliniquement sous forme d'un nodule ou d'une plaque cutanée ou sous cutanée. Le diamètre moyen est de 2 cm. Globalement bien limitée, non encapsulée et mobile par rapport aux plans sous jacents, indolore, non prurigineuse, le plus souvent de couleur rouge puis violacée, épargne l’épiderme et de croissance rapide [2, 5]. La présentation clinique du CCM n'est pas spécifique; le diagnostic positif repose sur l'analyse histologique d'une biopsie ou d'une exérèse de la tumeur, complétée par l'immuno- histochimie qui retrouve la présence de marqueurs neuro-endocrines. Les localisations tumorales possibles, par ordre décroissant, sont: la région cervico-céphalique (54%), les membres inférieurs (27%), les membres supérieurs et le tronc (19%) [1]. Les muqueuses sont généralement respectées, et exceptionnellement des adénopathies ou des métastases peuvent être révélatrices. De point de vue histopathologique, trois types d'architectures différentes sont décrits [2]: **le type trabèculaire:** est représenté par des amas de cellules sphériques avec un abondant cytoplasme et un noyau centré, l'invasion des tissus mous est la règle. Cette forme typique reste la moins fréquente; **Le CNEC à cellules intermédiaires** dont la structure rappelle le carcinome neuroendocrine pulmonaire. La dissociation des cellules simule un aspect lymphomateux. C'est la variété la plus fréquente; **le CNEC à petites cellules:** les formes pures ne peuvent être distinguées des carcinomes à petites cellules du poumon ou d'autres localisations.

Dans un but pronostique, il est recommandé de mesurer l’épaisseur tumorale et d’évaluer l'envahissement vasculaire [4]. L’étude immunohistochimique est d'un intérêt majeur car elle représente l’élément du diagnostic dans les cas difficiles en éliminant assez facilement surtout, un lymphome et un mélanome. Cependant, le véritable diagnostic différentiel se pose entre le CNEC primitif et une métastase d'un autre carcinome neuroendocrine en particulier d'origine bronchique [2]. Le CCM est une tumeur agressive à haut risque de dissémination lymphatique ou viscérale. Au moment du diagnostic, environ 20% des patients présentent un envahissement ganglionnaire local d'emblée, et 5% ont des métastases à distance [5].

### Bilan d'extension initial

Le bilan d'extension initial comprend un examen clinique complet de l'ensemble du tégument et de toutes les aires ganglionnaires, ainsi qu'un examen général à la recherche de métastases à distance. Il est recommandé de réaliser une échographie des aires ganglionnaires de drainage en raison du caractère extrêmement lymphophile du CCM, et un scanner thoraco- abdomino- pelvien dans tous les cas, ainsi que de la tête et du cou lorsque la tumeur primitive siège en région cervico- céphalique [4].

### Classification et pronostic

Le stade de la maladie a des implications pronostiques et conditionne la prise en charge thérapeutique; cependant plusieurs classifications ont été décrites dans la littérature et restent utilisées, ce qui est source de confusion dans l'interprétation des données de la littérature. La classification le plus souvent utilisée dans les études cliniques et les « guidelines » thérapeutiques est celle de l'American Joint Committee for Cancer (AJCC) proposée en 2010. Il s'agit d'un système de classification standardisé basé sur l'analyse de 4700 patients à partir du registre national du cancer américain, et qui prend en compte la taille de la tumeur primaire (< 2cm ou ≥ 2cm), l'existence d'une atteinte ganglionnaire régionale ou la présence de métastases à distance (Tableau 1) [5]. Selon les données actuelles de la littérature, les facteurs de mauvais pronostic clairement établis sont: le sexe masculin, l’âge inférieur à 60 ans, la taille supérieure à 2 cm, les critères histologiques (un index mitotique élevé avec un nombre de mitoses supérieur à 10 par champ, l'infiltration des vaisseaux lymphatiques, les formes à petites cellules ou à cellules intermédiaires), l'existence d'adénopathies ou de métastases (2). Le critère pronostique le plus important en terme de survie et de survenue de métastases à distance est la présence d'un envahissement ganglionnaire régional [5], puisque la survie à 5 ans dépasse 80% chez les malades qui en sont indemnes, tandis qu'elle est inférieure à 50% en cas d'atteinte ganglionnaire [5]. La procédure du ganglion sentinelle a été proposée dans le CCM depuis quelques années dans le but d'identifier précocement la présence de micrométastases ganglionnaires [5]. La présence de micrométastases lymphatiques dans le ganglion sentinelle est un facteur de mauvais pronostic avec environ 60% de rechute à 3 ans contre 20% seulement en l'absence de micrométastases [7]. Quelques cas de régression spontanée de la tumeur primitive ont été décrits en rapport avec une infiltration de la tumeur par des lymphocytes T induisant l'apoptose des cellules tumorales (5).

**Tableau 1:** Classification AJCC 2010 (American Joint Committee on Cancer: AJCC Cancer Staging Manual. 7th ed. New York, NY: Springer; 2010. p. 318-9)

T		N	M
Tx: tumeur primitive non évaluable T0: pas de tumeur primitive Tis: tumeur primitive in situ T1: tumeur primitive ≤ 2cm T2: tumeur primitive > 2cm mais ≤ 5cm T3: tumeur primitive > 5cm T4: tumeur primitive envahissant l'os/muscle/fascia/cartilage		Nx: ganglions de drainage non évaluables N0: pas de métastase ganglionnaire régionale cN0: ganglions non palpables cliniquement (a) cN1: ganglions cliniquement palpables (a) pN0: ganglions histologiquement négatifs pNx: ganglions non évalués histologiquement N1a: micrométastases (b) N1b: macrométastases (c) N2: métastases en transit (d)	Mx: métastases à distance non évaluable M0: pas de métastases à distance M1: métastases à distance (e) M1a: cutanées, des tissus mous ou ganglionnaires à distance M1b: pulmonaires M1c: autres métastases viscérales
**Stade**		**Critères**	
0	Tis	N0	M0
IA	T1	pN0	M0
IB	T1	cN0	M0
IIA	T2/T3	pN0	M0
IIB	T2/T3	cN0	M0
IIC	T4	N0	M0
IIIA	Tous T	N1a	M0
IIIB	Tous T	N1b/N2	M0
IV	Tous T	Tous N	M1

(a) N0 signifie absence d'atteinte ganglionnaire clinique et/ou histologique. Le ganglion pathologique clinique peut être détecté à l'inspection, à la palpation ou par un examen radiologique; cN0 est utilisé uniquement pour les patients n'ayant pas eu de biopsie-exérèse ou curage ganglionnaire avec analyse histologique.

(b) Les micrométastases sont identifiées par la technique du ganglion sentinelle.

(c) Les macrométastases correspondent aux ganglions pathologiques cliniquement, dont la nature pathologique est confirmée histologiquement.

(d) Une métastase en transit est une tumeur distincte de la tumeur primitive et localisée soit entre la lésion primitive et le territoire ganglionnaire de drainage.

(e) M1 a-c sont inclus dans le même groupe parce qu'il n'existe pas actuellement de données en faveur de sous-groupes pronostiques en fonction de la localisation des métastases.

### Traitement

En raison de la rareté du carcinome à cellules de Merkel, sa prise en charge ne fait pas l'objet d'un consensus. Des recommandations pour la pratique ont été publiées aux Etats-Unis, et en Allemagne [3]. Les accords entre les deux recommandations ont été récemment validés en France, par le groupe de cancérologie cutanée de la société française de dermatologie, qui a proposé un guide de prise en charge [4].

### Le traitement chirurgical

C'est la base du traitement dans la plupart des cas, il intéresse à la fois le site de la tumeur primitive et l'aire ganglionnaire de drainage. Il faut distinguer plusieurs situations: ***En l'absence d'atteinte ganglionnaire et de métastase à distance, clinique ou radiologique***: s'il n'y a pas encore eu de reprise ou d'exérèse élargie, il est recommandé de réaliser l'exérèse ou la reprise large de la tumeur, avec une marge cutanée latérale cliniquement saine de 2 à 3 cm (c'est le cas pour notre patient), quelle que soit la taille de la tumeur, voire de 1 cm lorsque la localisation rend difficile de larges exérèses. Cette exérèse doit aller en profondeur jusqu'au fascia, qui sera conservé en l'absence d'invasion clinique. La reconstruction est soit immédiate ce qui est souvent possible, soit en seconde intention dans les formes étendues, et elle privilégiera des techniques simples facilitant la surveillance ultérieure (suture directe, greffe de peau totale, etc.) au détriment des lambeaux complexes. Si la topographie le permet et après repérage scintigraphique, il est recommandé de pratiquer la biopsie-exérèse du ganglion sentinelle pour étude anatomopathologique, dans le même temps chirurgical quelle que soit la taille de la tumeur primitive. En effet, l'analyse du ganglion sentinelle permet d'affiner le stade du CCM et de guider le traitement complémentaire. Lorsque l'analyse histologique du ganglion sentinelle est positive, un curage ganglionnaire complémentaire est indiqué, malgré l'absence d’étude démontrant que cette attitude est associée à un allongement de la survie. Si la reprise ou l'exérèse élargie a déjà eu lieu, il n'est pas recommandé de pratiquer l'exérèse du ganglion sentinelle a posteriori, ni d'effectuer un curage ganglionnaire à visée prophylactique; ***En cas d'atteinte ganglionnaire clinique ou radiologique, et après avoir vérifié l'absence de métastase viscérale à distance***: un curage ganglionnaire est indiqué, en association à l'exérèse large, comme précédemment décrit, de la tumeur primitive. Globalement, la lymphadenectomie systématique est discutée. Néanmoins, elle s'impose lorsque la taille de la tumeur dépasse 2 cm et lorsque le nombre de mitoses est supérieur à 10 par champ ou lorsqu'il s'agit d'un type à petites cellules ou à cellules intermédiaires. La lymphadenectomie est, par contre, impérative en présence d'adénopathies palpables [2].

### La radiothérapie

L'utilisation de la radiothérapie, en complément de la chirurgie, semble diminuer le risque de récidive locorégionale et améliorer la survie globale. La radiothérapie externe, à titre adjuvant, est donc systématiquement recommandée sur le site tumoral primitif, dans les localisations cervico-faciales où la résection large n'est pas toujours possible et lorsque l'envahissement ganglionnaire est confirmé histologiquement, c'est le cas pour notre patient [2, 4]. Un traitement par radiothérapie exclusive peut être envisagé dans le cas de tumeurs dont l'exérèse n'est pas réalisable. Si la biopsie du ganglion sentinelle n'a pas pu être réalisée, l'indication de la radiothérapie sur l'aire ganglionnaire de drainage doit être discutée; elle est conseillée pour les tumeurs volumineuses (> 2 cm de diamètre). Dans le cas où la biopsie du ganglion sentinelle a été réalisée, si l'analyse histologique est négative, il n'y a pas, a priori, d'indication à effectuer une radiothérapie prophylactique sur le territoire de drainage. Cela peut, néanmoins, être discuté en réunion de concertation pluridisciplinaire au cas par cas, en fonction des facteurs de mauvais pronostic associés. Si un curage a été réalisé (atteinte ganglionnaire clinique ou ganglion sentinelle positif), la radiothérapie est recommandée sur l'aire ganglionnaire opérée, c'est le cas chez notre patient. La radiothérapie peut être proposée comme traitement palliatif de certains sites métastatiques, compte tenu de la radiosensibilité de la tumeur [4].

### La chimiothérapie

En l'absence de métastases à distance, la chimiothérapie adjuvante n'améliore pas la survie des patients [8]. En cas de dissémination métastatique, la chimiothérapie reste l'arme thérapeutique de choix (2), bien qu'il n'existe pas de données montrant une amélioration du pronostic à ce stade de la maladie [9, 10] où une prise en charge palliative exclusive peut être discutée. Les deux protocoles, habituellement utilisés, reposent sur l'association carboplatine-étoposide ou cyclophosphamide-doxorubicine-vincristine (4).

### Surveillance

D'après les recommandations élaborées par le groupe de cancérologie cutanée de la Société française de dermatologie (4), il est recommandé de surveiller étroitement les patients atteints d'un CCM une fois la prise en charge thérapeutique initiale réalisée. L'autosurveillance à la recherche d'un autre cancer cutané, d'une récidive locale, en transit ou ganglionnaire doit être proposée aux patients. La fréquence de la surveillance doit être discutée en fonction des facteurs de risque associés. Mais, Il semble raisonnable selon le même groupe, de proposer au minimum une surveillance clinique trimestrielle les deux premières années, puis tous les 6 mois pour une durée totale de 5 ans. L'examen clinique a pour but la recherche d'une récidive locale, en transit, ganglionnaire ou à distance. L’échographie des aires ganglionnaires de drainage, de la cicatrice d'exérèse et à la recherche de métastases cutanées ou sous-cutanées peut être proposée en complément de l'examen clinique en raison de la fréquence des récidives ganglionnaires et de la possibilité d'une nouvelle sanction chirurgicale ou radiothérapeutique. Une TDM corps entier peut être proposée en option en fonction de l'atteinte initiale: plus volontiers en cas d'atteinte ganglionnaire, pas obligatoirement en absence d'atteinte ganglionnaire. L’évolution peut être marquée par la survenue de métastases dans la moitié des cas. Les sites métastatiques habituels sont: les ganglions, le foie, les tissus mous et plus rarement le poumon et le système nerveux central. Environ 35% des CNEC présentent une récidive locale dans l'année qui suit le traitement surtout lorsqu'il existe d'emblée des métastases (2). Globalement le pronostic est mauvais et le taux de survie à cinq ans allant de 30 à 64% (4).

## Conclusion

Le carcinome neuro-endocrine cutané primitif (CNEC) est une tumeur cutanée rare, agressive et dont l'incidence est en augmentation franche, essentiellement en raison du vieillissement de la population. La prise en charge diagnostique et thérapeutique de cette tumeur n'est pas consensuelle en absence d’études randomisées prospectives de grande envergure. Le pronostic reste sombre malgré les progrès thérapeutiques actuels.
